# Retraction: SOX2 Enhances the Migration and Invasion of Ovarian Cancer Cells via Src Kinase

**DOI:** 10.1371/journal.pone.0295163

**Published:** 2023-11-28

**Authors:** The PLOS ONE Editors

Following the publication of this article [1], concerns were raised regarding Figs 4,6 and 7. Specifically:

- In Fig 4A the invasion assay vector and SOX2 panels appear similar to the invasion assay SOX2 and SOX2/si-Src panels found in Fig 7A respectively.
- In Fig 6B the following bands appear similar:
  ○ The H08910 cell MMP-2 panel and the Skov3 MMP-2 panel when rotated through 180 degrees.
  ○ The HO8910-pm NC p-P130cas (Y410) band and the Skov3 NC P130cas band when the latter is horizontally flipped.
  ○ The HO8910 SOX2 P130cas band and the Skov3 si-SOX2 P130cas band.
  ○ The HO8910 Vector MMP-9 band and the HO8910-pm si-SOX2 MMP-9 band.
  ○ The HO8910-pm NC GAPDH band and the Skov3 si-SOX2 GAPDH band.
- In Fig 7B the following bands appear similar to bands found in Fig 6B:
  ○ The SOX2/si-SRC -/- p-P130cas (Y410) band and the HO8910 SOX2 P130cas band.
  ○ The SOX2/si-SRC +/- p-P130cas (Y410) band and the HO8910-pm NC p-P130cas (Y410) band.
  ○ The SOX2/si-SRC +/+ p-P130cas (Y410) band and the HO8910-pm si-SOX2 p-P130cas (Y410) band.
- HO8910 cells are suspected of being HeLa derivatives rather than an ovarian cancer cell line [2].

The authors stated that the similarity of panels in Fig 4A and 7A were due to errors made in the preparation of figures and provided images described as underlying data for these panels and a corrected version of Fig 4A. The authors provided some of the original data for Figs 6B and 7B but stated certain parts of the data were unavailable in addition to providing replication data. Due to the extensive nature of the concerns regarding Western blots in this article, the concerns regarding these figures can only be resolved via provision of original underlying data in its entirety for the published panels, which was not made available for editorial review. As such the PLOS Editors remain concerned about Figs 6B and 7B. The authors stated that concerns regarding the HO8910 cells were not known at the time of the study but did not provide data characterising the cells in question as ovarian cancer.

In light of the concerns affecting multiple figure panels that question the integrity and reliability of these data, the *PLOS ONE* Editors retract this article.

XW did not agree with the retraction. XJ, JC, DY, ZZ, QW, XX, and YF either did not respond directly or could not be reached.
